# Genome Sequence of the Estuarine *Synechococcus* sp. Strain NB0720_010

**DOI:** 10.1128/mra.00151-22

**Published:** 2022-07-13

**Authors:** Campbell A. MacKenzie, Marcia F. Marston, Javier F. Tabima, Nathan A. Ahlgren

**Affiliations:** a Biology Department, Clark University, Worcester, Massachusetts, USA; b Department of Biology, Marine Biology and Environmental Science, Roger Williams University, Bristol, Rhode Island, USA; University of Southern California

## Abstract

Marine *Synechococcus* spp. are unicellular cyanobacteria widely distributed in the world’s oceans. We report the complete genome sequence of *Synechococcus* sp. strain NB0720_010, isolated from Narragansett Bay, Rhode Island. NB0702_10 has several large (>3,000-amino acid) protein-coding genes that may be important in its interactions with other cells, including grazers in estuarine habitats.

## ANNOUNCEMENT

Marine *Synechococcus* spp. are photosynthetic bacteria found in the surface waters of nearly every marine habitat on the planet and are estimated to contribute to ~17% of annual net marine primary productivity (1). While there are many genomes available for open-ocean *Synechococcus* spp. (2, 3), there has been limited genome sequencing of coastal and estuarine isolates (4). The genome sequence of *Synechococcus* sp. strain NB0720_010, isolated from Narragansett Bay and belonging to the marine *Synechococcus* subcluster 5.2 clade CB5, adds to our knowledge of genomic diversity in nearshore and estuarine *Synechococcus* populations.

Strain NB0720_010 was isolated from water collected on 28 July 2020 at 10:15 a.m. on an incoming tide in Narragansett Bay from a dock at 41°38′59.5812″N, 71°15′24.2038″W. NB0720_010 was isolated by pour plating using the methods outlined in reference 5 with modifications. Sample seawater was prefiltered through a 1-μm filter, mixed with an equal volume of 2× PRO99 medium (5) with 0.64% molten (~33°C) low-melting-point agarose, and immediately poured onto a 0.7% agarose PRO99 base layer. The plate was incubated at 22°C with a 14:10-h light/dark illumination cycle at ~20 μE m^−2^ s^−1^ for several weeks until a single colony was picked and transferred to Pro99 liquid medium. After a few transfers to liquid medium, DNA was extracted from a late-exponential-phase culture using a phenol-chloroform extraction protocol (6, 7).

Illumina shotgun sequencing performed by the Microbial Genome Sequencing Center (Pittsburgh, PA; library preparation using the Nextera XT kit following the manufacturer’s directions; sequencer, NextSeq 550) yielded 2,649,963 paired-end 151-bp reads. Default parameters were used for all software unless otherwise specified. The reads were trimmed using Trimmomatic v0.38 (8) with the following settings: ILLUMINACLIP:TruSeq3-PE-2.fa:2:30:10, LEADING:10, TRAILING:10 SLIDINGWINDOW:4:15 MINLEN:50. Long-read Nanopore sequencing was performed in-house using DNA from the same culture but extracted from a different inoculum (library preparation using the SQKLSK-110 ligation kit following the manufacturer’s directions; Flongle flow cell R9.4.1), and this yielded 874,048 reads (mean length, 255 bp; *N*_50_, 69,887 bp; base calling using Guppy v5.0.11 + 2b6dbff; Oxford Nanopore). Assembly of the Illumina and Nanopore reads using Unicycler v0.4.9b (9) generated 630 contigs (*N*_50_, 8 kb; total length, 18 Mb). Among these was a 2,410,448-bp circular contig with 63% GC content and 97.1% average nucleotide identity (calculated using FastANI [10]) to estuarine *Synechococcus* sp. strain CB0205 (GenBank accession number GCA_000179255.1) (11, 12), belonging to subcluster 5.2 clade CB5, and thus, this contig was deemed to be the complete genome of *Synechococcus* sp. NB0720_010. It was annotated using the NCBI Prokaryotic Genome Annotation Pipeline v5.3 (13), yielding 2,530 protein-coding genes, 3 complete rRNA operons (5S, 16S, and 23S), and 46 tRNAs.

NB0720_010 contains several putative type II antitoxin/toxin protein-coding genes. Most of these have homologs (determined using a blastp search [14]; E value < 1e–10) in other estuarine but not open-ocean *Synechococcus* isolate genomes, consistent with previous observations (15). NB0720_010 also contains four very large (>3,000 amino-acid [aa]), or “giant,” protein-coding genes that all have repeated motifs possessing similarity to cadherin-like or RTX toxin domains (Table 1). Such giant proteins often occur in marine *Synechococcus* genomes (19), including SwmB in *Synechococcus* sp. WH8102 (10,791 aa), which is involved in swimming motility and resisting protistan predation (19–21). Similar to SwmB, we suggest that these giant proteins in NB0720_010 may be important for defense and/or competition, which may be crucial in estuarine waters, which typically have higher cell densities than open-ocean regions.

**TABLE 1:** Characteristics of the four very large (>3,000-aa) proteins in *Synechococcus* sp. strain NB0720_010

Locus tag	Protein length (aa)	Similarity to other giant proteins in *Synechococcus* isolate genomes^*a*^	Domain description
LY254_04120	10,749	30% identity over 44% of the protein to SynWH8101_0818 (4,083 aa; QBE68408.1) in *Synechococcus* sp. strain WH 8101 (16)	Several repeated domains (~100 aa long) with similarity to cadherin-like domains (pfam17803, pfam17892), bacterial Ig^*b*^ domains (pfam17963), and repeats (TIGR01965) found frequently in *Vibrio*, *Colwellia*, *Bradyrhizobium*, and *Shewanella* (VCBS) members
LY254_03050	4,609	30% identity over 82% of the protein to EVJ50_06065 (11,376 aa; QEY31874.1) in *Synechococcus* sp. strain RSCCF101 (17)	Several repeated domains (DUF5801, pfam19116)
		36% identity over 82% of the protein to Legionella pneumophila toxin protein RtxA (STX70670.1)	
LY254_05360	3,090	No hits to isolate genomes but several to short (<700-aa) proteins from *Synechococcaceae* freshwater metagenomic assembled genomes (18)	Several repeated cadherin-like domains (pfam17892) and a C-type lectin-like domain (cd03603)
LY254_11890	3,087	96% identity over 100% of the protein to KJJ24_00540 (3,087 aa; QVV67743.1) in *Synechococcus* sp. strain LA31 (Narragansett Bay isolate) (4)	RTX toxin domain repeats (NF033203, NF033943) and an RTX toxin-related domain (COG2931)

a Top hit(s) from a *Synechococcus* isolate from a blastp search (13) against NCBI’s nonredundant (nr) database with an E value of <1e–5. The lengths and GenBank accession numbers of the protein hits are listed in parentheses.

b Ig, immunoglobin.

### Data availability.

*Synechococcus* sp. NB0720_10 is available from Nathan A. Ahlgren upon request. The sequence data are available at NCBI under BioProject accession number PRJNA793027, including the raw reads (SRA accession numbers SRR18042579 and SRR18042580) and the assembled genome (GenBank accession number CP090898).
